# Support System of Cystoscopic Diagnosis for Bladder Cancer Based on Artificial Intelligence

**DOI:** 10.1089/end.2019.0509

**Published:** 2020-03-17

**Authors:** Atsushi Ikeda, Hirokazu Nosato, Yuta Kochi, Takahiro Kojima, Koji Kawai, Hidenori Sakanashi, Masahiro Murakawa, Hiroyuki Nishiyama

**Affiliations:** ^1^ Department of Urology, University of Tsukuba Hospital, Tsukuba, Japan.; ^2^ Artificial Intelligence Research Center, National Institute of Advanced Industrial Science and Technology, Tsukuba, Japan.; ^3^ Department of Intelligent Interaction Technologies, Graduate School of System and Information Engineering, University of Tsukuba, Tsukuba, Japan.; ^4^ Department of Urology, Faculty of Medicine, University of Tsukuba, Tsukuba, Japan.

**Keywords:** artificial intelligence, bladder cancer, cystoscopy, transfer learning, deep learning

## Abstract

***Introduction:*** Nonmuscle-invasive bladder cancer has a relatively high postoperative recurrence rate despite the implementation of conventional treatment methods. Cystoscopy is essential for diagnosing and monitoring bladder cancer, but lesions are overlooked while using white-light imaging. Using cystoscopy, tumors with a small diameter; flat tumors, such as carcinoma *in situ*; and the extent of flat lesions associated with the elevated lesions are difficult to identify. In addition, the accuracy of diagnosis and treatment using cystoscopy varies according to the skill and experience of physicians. Therefore, to improve the quality of bladder cancer diagnosis, we aimed to support the cystoscopic diagnosis of bladder cancer using artificial intelligence (AI).

***Materials and Methods:*** A total of 2102 cystoscopic images, consisting of 1671 images of normal tissue and 431 images of tumor lesions, were used to create a dataset with an 8:2 ratio of training and test images. We constructed a tumor classifier based on a convolutional neural network (CNN). The performance of the trained classifier was evaluated using test data. True-positive rate and false-positive rate were plotted when the threshold was changed as the receiver operating characteristic (ROC) curve.

***Results:*** In the test data (tumor image: 87, normal image: 335), 78 images were true positive, 315 true negative, 20 false positive, and 9 false negative. The area under the ROC curve was 0.98, with a maximum Youden index of 0.837, sensitivity of 89.7%, and specificity of 94.0%.

***Conclusion:*** By objectively evaluating the cystoscopic image with CNN, it was possible to classify the image, including tumor lesions and normality. The objective evaluation of cystoscopic images using AI is expected to contribute to improvement in the accuracy of the diagnosis and treatment of bladder cancer.

## Introduction

Spatial and temporal development at multiple sites is a clinical characteristic of bladder cancer, and the frequencies of its presence at multiple intravesical sites at the time of diagnosis and intravesical recurrence after complete endoscopic resection of visible lesions are high.^1^ The standard treatment for nonmuscle-invasive bladder cancer (NMIBC) is endoscopic transurethral resection of the bladder tumor, but it has been reported that intravesical recurrence occurs within 2 years after operation in ∼50% of cases.^2^ Moreover, the recurrence rates at the first cystoscopy after TUR-BT vary widely among institutions, and the diagnostic accuracy also varies according to differences in the urologist's skill and experience.^3,4^

Cystoscopy is essential for diagnosing bladder cancer and observing the course, but lesions are overlooked during observation under white-light imaging (WLI) in 10%–20% of cases.^5,6^ The reported sensitivity and specificity of diagnosis under WLI are ∼60% and 70%,^7^ respectively, and it is difficult to identify the expansion of flat lesions accompanying flat-type tumors and elevated lesions of tumors with a small diameter and carcinoma *in situ*. Endoscopic techniques, such as narrow band imaging (NBI) and photodynamic diagnosis (PDD), have been developed to improve visibility of bladder cancers, and their usefulness has been demonstrated^8–12^; however, WLI is still the primary method of observation.

Imaging diagnosis utilizing artificial intelligence (AI) has recently been developed in the field of medicine. Endoscopic imaging-based diagnosis using AI has been clinically applied in the field of gastroenterology, but its application to the field of urology was initiated only recently.^13–16^ In this study, to improve the quality of bladder cancer diagnosis, we aimed to objectively evaluate normal and tumor images obtained by white-light cystoscopy using AI.

## Materials and Methods

### Preparation of training and test image sets

**FIG. 1.**
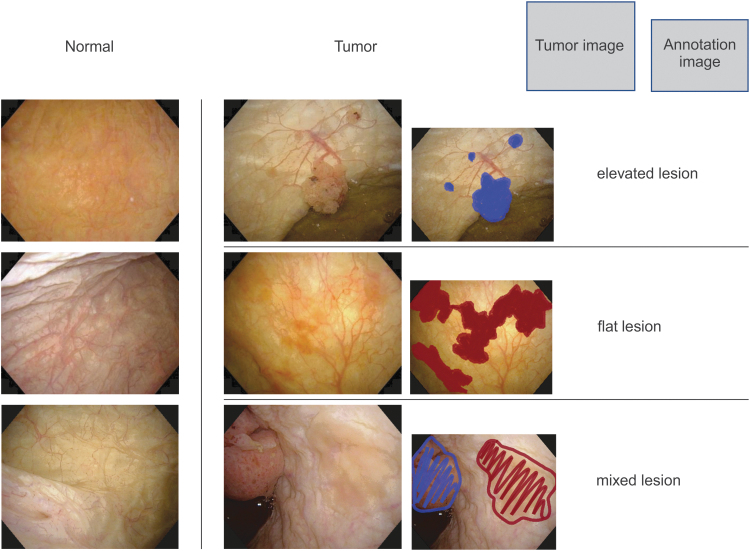
Sample of cystoscopic and annotation images.

Endoscopic images of the bladder acquired at the University of Tsukuba Hospital between February 2017 and July 2018 were used. For endoscopic images, still images acquired using a flexible endoscope (CYF-VHA; Olympus Medical System, Co., Ltd., Tokyo, Japan) at the outpatient clinic were used. For the endoscopic image of the bladder, 2102 images were obtained with the TIFF file of 1350 × 1080 pixels by white light. Images with urine turbidity and images that were out of focus were excluded. One dataset included 431 images of tumors, and one urologist marked the sites judged to be a tumor using two patterns, namely, elevated and flat lesions, regardless of whether the tumor was malignant or benign. The actual judgment was also confirmed with the pathologic results as ground truth. The other dataset contained 1671 normal images, judged by the same doctor as not showing tumor lesions based on cystoscopy performed 1637 times around the same period. Figure 1 presents the normal image, tumor image, and a sample of the annotation data.

### Constructing a convolutional neural network and outcome measures

**FIG. 2.**
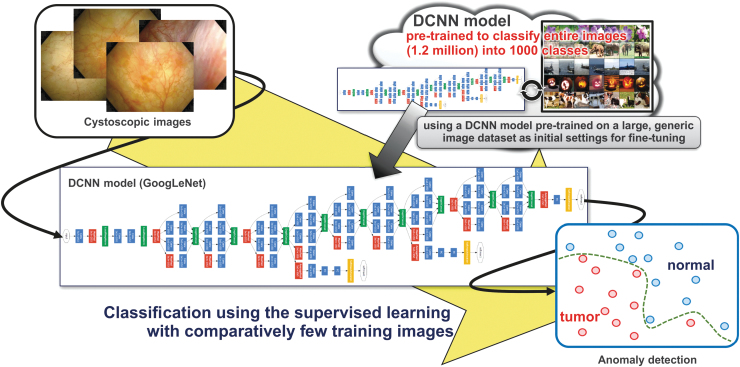
A method based on transfer learning with pretrained convolutional neural networks.

The cystoscopic images were evaluated using the convolutional neural network (CNN) model based on GoogLeNet^17^ with transfer learning. GoogLeNet is a CNN model that has been awarded the first prize at ImageNet large scale visual recognition challenge 2014, and developed by training through the task of 1000-class classification using ImageNet^18^ dataset consisting of 1.2 million natural images. In the proposed method, network parameters of this pretrained CNN model were transferred to the initial network parameters to learn cystoscopic images additionally in supervised learning (Fig. 2). Then, all network parameters of this CNN were fine-tuned using the Adam algorithm^19^ to identify lesions and normal images. The training dataset was augmented by adding new data that were generated by rotating and blurring the images, which were randomly chosen from the original image. Using this augmentation, the numbers of tumor and normal images in the training dataset were obtained in the ratio of 1:1. All endoscopic images of the bladder were randomly divided into two (the training and test) datasets with a ratio of 8:2. The learning rate was 1e-5, and the epoch count was 150. Whether the image is an endoscopic image of the bladder containing lesions overall or a normal image could be distinguished by performing learning by GoogLeNet as a classifier using multilayer perceptron in the discriminator with set learning data (tumor images: 344, normal images: 1336) and test data (tumor images: 87, normal images: 335).

### Statistical analysis

The programming language Python was used throughout the experiment. We used “Chainer,” which is a deep learning framework to model and learn CNN. When evaluating, we used the machine learning library “scikit-learn” to calculate the confusion matrix and receiver operating characteristic (ROC) curve. The classifier was trained to output 1 if the image showed a tumor and 0 if the image was normal using training data. The performance of the classifier was evaluated using test data. True-positive rate and false-positive rate were plotted when the threshold was changed from 0 to 1 as the ROC curve. The area under the curve (AUC) was calculated from the ROC curve. In addition, we calculated the maximum Youden index (YI), sensitivity, and specificity.

### Ethics

This study was approved by the Ethics Committee of the University of Tsukuba Hospital (no. H28-235) and National Institute of Advanced Industrial Science and Technology (no. Hi2016-224). An opt-out approach was used when obtaining consent from the patients before the study participation.

## Results

Table 1 presents the summary of the patients recruited and the tumor images. A total of 124 bladder endoscopies were performed on 109 patients (97 men and 12 women) during the study period. Among the 431 images obtained, 1 (0.2%) showed a papilloma and a benign tumor. However, the remaining images showed urothelial carcinoma. By T classification, 15 (3.5%) images were T2 muscle-invasive bladder cancer, and the rest (96.3%) were Ta, Tis, and T1 NMIBC. The level of malignancy was of low and high grades at 45.3% and 54.5%, respectively. The annotation data of the lesion image showed an elevated lesion for 265 (61.5%) images, flat lesion for 76 (17.6%), and a mixture of raised and flat lesions for 90 (20.9%). The proportion of images with a tumor size occupying <10%, 10%–50%, and >50% of the overall image was 10.2%, 56.9%, and 32.9%, respectively.

**Table 1.** Patient (*n* = 109) and Tumor Characteristics in the Image Set (Four Hundred Thirty-One Images)

	n	%
Male	97	90.0
Female	12	10.0
Age, years		
Median (interquartile range)	74 (64.5–77)	
Tumor form		
Elevated lesion	265	61.5
Flat lesion	76	17.6
Mixed lesion	90	20.9
Pathology analysis of tumor		
Benign, papilloma	1	0.2
Urothelial carcinoma	430	99.8
TNM stage		
Ta	329	76.5
T1	38	8.8
Ta +1	16	3.7
Tis	21	4.9
Ta + is	11	2.6
T2	15	3.5
Grade^a^		
Low grade	195	45.3
High grade	235	54.7
Tumor size		
Proportion of the overall image occupied by the lesion		
>10%	44	10.2
10%–50%	245	56.9
>50%	142	32.9

^a^ 1973 World Health Organization classification.

CIS = carcinoma *in situ*; TNM = tumor–node–metastasis.

**FIG. 3.**
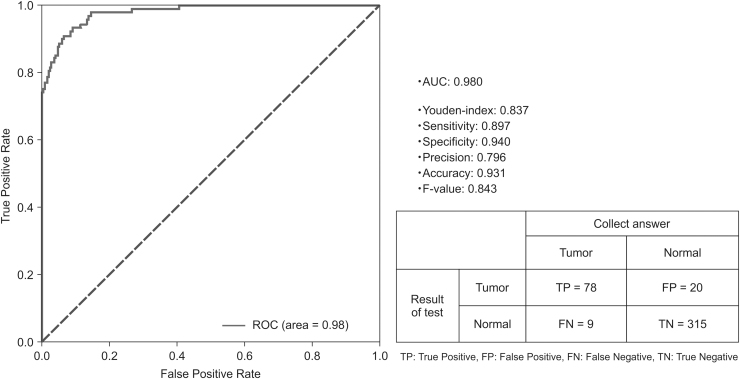
MLP ROC curve. MLP = multilayer perceptron; ROC = receiver operating characteristic.

**FIG. 4.**
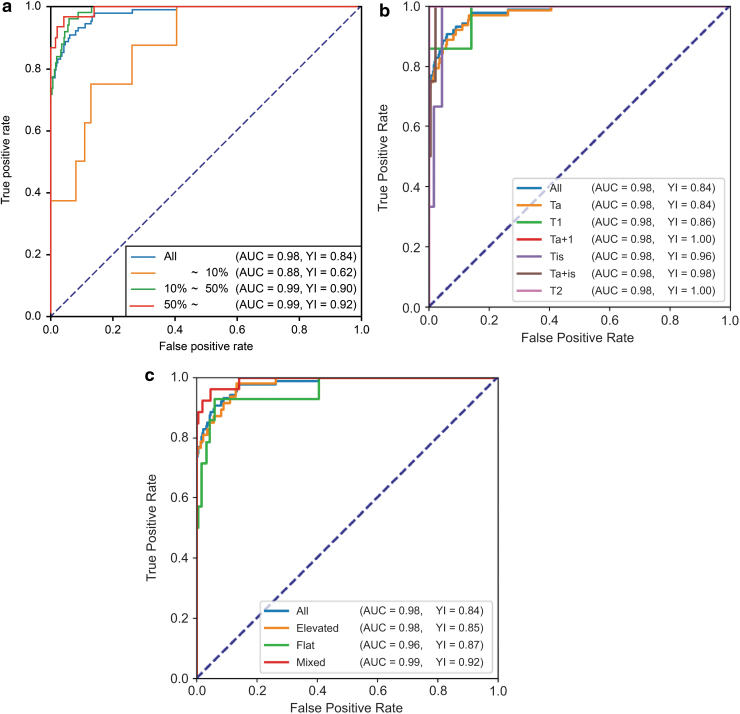
**(a)** MLP ROC curve based on the proportion of lesions in the image. **(b)** MLP ROC curve based on the T stage of tumor. **(c)** MLP ROC curve based on the form of tumor.

In the ROC curve (Fig. 3), AUC was 0.98, maximum YI was 0.837, sensitivity was 89.7%, and specificity was 94.0%. In the test data (tumor image: 87, normal image: 335), 78 images were true positive, 315 true negative, 20 false positive, and 9 were false negative. Of the nine images that were false negative, six were early discovered raised lesions that included Ta tumors. In addition, two images showed flat lesions of Ta tumors, and one image was a T1 tumor for which annotation of a mixed raised and flat lesion was performed. Of the nine images, eight images showed a small lesion that occupied <10% of the overall image. Figure 4a shows the ROC curve according to the proportion of lesions in the image. In <10%, 10%–50%, and >50% of the images, AUC and YI were 0.88 and 0.62, 0.88 and 0.90, and 0.88 and 0.92, respectively. Figure 4b shows the ROC curve according to the T stage of tumor. Ta, T1, Ta +1, Tis, Ta + is, and T2, AUC and YI were 0.98 and 0.84, 0.98 and 0.86, 1.00 and 1.00, 0.98 and 0.96, 0.99 and 0.98, and 1.00 and 1.00, respectively. Figure 4c shows the ROC curve according to the form of tumor. Elevated, flat, and mixed AUC and YI were 0.98 and 0.85, 0.96 and 0.87, and 0.99 and 0.92, respectively.

## Discussion

Regarding the mechanism of intravesical recurrence of NMIBC, dissemination of tumor cells, expansion of precancerous lesions, and overlooking of micro-disseminated lesions (daughter tumors) are considered rather than the *de novo* development. Therefore, it is of utmost importance to ensure the resection of disseminated lesions (daughter tumors), carcinoma *in situ*, and expansion of precancerous lesions that are difficult to observe without overlooking during TUR-BT, in addition to the main tumor. To improve the quality of bladder cancer diagnosis, we have proposed an objective evaluation method of cystoscopic imaging using AI, which allowed us to discriminate normal and tumor images with high accuracy.

AI has recently been applied in the medical field, such as in the diagnosis of pulmonary nodules on computed tomography,^20^ diagnosis of skin cancer,^21^ classification of retinal lesions based on fundus photographs,^22^ endoscopic imaging diagnosis, detection of gastric cancer,^13^ evaluation of *Helicobacter pylori* infection of the stomach,^14^ classification of colon polyps,^15^ and automatic classification of regions in the upper gastrointestinal endoscopic images.^23^ Diagnosis of prostate cancer lesions on prostate magnetic resonance imaging has been attempted in the field of urology,^24^ but only one study from the urologic endoscopy field has been reported.^25^

In this study, using white-light endoscopic images, favorable results of diagnosis were achieved for tumors with high sensitivity and specificity. It was clarified that through learning of ∼1700 cystoscopic images, normal and tumor lesions can be classified by distinguishing using transfer learning based on CNN. In general, learning of AI requires millions of large amount and high-quality data, but it was possible to detect tumor lesions in cystoscopic images even when learning as few as several thousand images through transfer learning, whereby the features of cystoscopic images are extracted from a deep learning model constructed using 1.2 million general images. It is suggested that transfer learning adopting a deep learning model to extract features facilitates an adjunctive diagnosis by machine learning and increases the quality of diagnosis and treatment of bladder cancer although a large volume of imaging data for learning cannot be prepared, such as the collection of medical images at single institutions. Transfer learning used in this study is similar to the fact that humans recognize normal and abnormal states by learning, that is, we observe various general images as we grow from birth, and after becoming a medical student and then physician, we learn endoscopic images through training, thereby acquiring the skills to make a diagnosis.

We do not consider that this achievement replaces diagnoses made by urologists. If an automatic visual detection method for cystoscopic images can be established using this method, it may support doctors to make a diagnosis based on not only still images but also videos and real-time cystoscopy, which leads to accurate identification of the range of the tumor and reduction of the diagnostic miss rate, increasing the quality of medical care.

The accuracy of annotating images of lesions, based on learned data, is a limitation of this study. At present, one urologist prepared all annotation data, so that the diagnoses of lesions are consistent, but the possibility of overlooking a lesion cannot be ruled out. In addition, the method on how the test data are classified as tumor images or normal images by multiple doctors has not been verified. Moreover, annotation data, such as inflammation-induced changes in the bladder mucosa, were not learned because these were not prepared. The difficulty of learning may be increased by the turbidity of urine, being out of focus, and whether the image is acquired from a near or distant site.

If a tumor is reflected in a cystoscopic image, abnormalities can be identified. When doctors recognize a tumor, they examine it closely. However, if they do not recognize the tumor, they would not examine it closely and may therefore overlook it. These issues may be solved by diagnostic support using AI. In addition, for tumor lesions that are likely to be overlooked by WLI, learning of NBI, PDD, and high-quality endoscopic images such as 4K may reduce these and lead to a higher diagnostic accuracy.

At present, this study has resulted in a low diagnostic accuracy for small lesions that occupied <10% of the overall image. This may be because of insufficient learning by the AI system as only ∼10% of the images used contained small lesions. These small lesions are also likely to be missed by urologists. In general, the performance of an AI system is dependent on the amount and quality of data used for learning. Although the AI system was able to extract and objectively assess features in cystoscopy images, new algorithms and additional images representing a variety of lesions are needed to improve its accuracy.

## Conclusion

This study proposed a support system for the cystoscopic diagnosis of bladder cancer based on AI. The proposed AI system, as an initial support system of cystoscopic diagnosis, was based on a pretrained CNN model with ImageNet and was fine-tuned to learn images from cystoscopy performed 1637 times additionally in supervised learning. We demonstrated that our AI system is capable of classifying tumor lesions and normality with high accuracy for 431 test images from 109 patients. For clinical application, the proposed AI system would be further verified when in clinical use, so as to further develop it as a solution for diagnosis support. The objective evaluation of cystoscopic images using AI is expected to contribute to improvement in the accuracy of the diagnosis and treatment of bladder cancer.

## Ethical Standards

All procedures followed were in accordance with the ethical standards of the responsible committee on human experimentation (institutional and national) and with the Declaration of Helsinki of 1964 and later versions. Informed consent or substitute for it was obtained from all patients for being included in the study.

## Abbreviations Used

AI: artificial intelligence
AUC: area under the curve
CIS: carcinoma *in situ*
CNN: convolutional neural network
DCNN: deep convolutional neural network
MLP: multilayer perceptron
NBI: narrow band imaging
NMIBC: nonmuscle-invasive bladder cancer
PDD: photodynamic diagnosis
ROC: receiver operating characteristic
TNM: tumor–node–metastasis
WLI: white-light imaging
YI: Youden index
